# Functional outcome of patients with prolonged hypoglycemic encephalopathy

**DOI:** 10.1186/s13613-017-0277-2

**Published:** 2017-05-22

**Authors:** Guillaume Barbara, Bruno Mégarbane, Laurent Argaud, Guillaume Louis, Nicolas Lerolle, Francis Schneider, Stéphane Gaudry, Nicolas Barbarot, Angéline Jamet, Hervé Outin, Sébastien Gibot, Pierre-Edouard Bollaert

**Affiliations:** 1 Service de Réanimation médicale, Hôpital Central, CHU de Nancy, Av. de Lattre de Tassigny, 54035 Nancy Cedex, France; 2 Service de Réanimation Médicale et Toxicologique, CHU Lariboisière, INSERM U1144, Université Paris Diderot, Paris, France; 3 Service de Réanimation Médicale, Hôpital Edouard Herriot, Lyon, France; 4 Service de Réanimation Polyvalente, CHR de Metz-Thionville, Metz, France; 5 Service de Réanimation Médicale et de Médecine Hyperbare, CHU et Université d’Angers, Angers, France; 6 Service de Réanimation Médicale, Hôpital de Hautepierre, HUS, Fédération de Médecine Translationnelle de Strasbourg et U1121 INSERM, Université de Strasbourg, Strasbourg, France; 7 Service de Réanimation Médico-Chirurgicale, Hôpital Louis Mourier, Colombes, France; 8 UMRS 1123, Univ Paris Diderot, Paris, France; 9 Service de Réanimation Polyvalente, CH de St Brieuc, St Brieuc, France; 10 Service de Réanimation Médicale, CHU de Poitiers, Poitiers, France; 11 Service de Réanimation médico-chirurgicale, CHI de Poissy-Saint Germain en Laye, Poissy, France

**Keywords:** Hypoglycemia, Hypoglycemic encephalopathy, Patient outcome assessment, Intensive care units, Care withdrawal, Brain imaging

## Abstract

**Background:**

Little is known about the causes, clinical course and long-term outcome of comatose patients with prolonged hypoglycemic encephalopathy.

**Methods:**

In a multicenter retrospective study conducted in patients hospitalized from July 1, 2004, to July 1, 2014, we investigated functional long-term prognosis and identified prognosis factors of patients admitted in an intensive care unit (ICU) with prolonged neurological manifestations related to hypoglycemia. Eligible patients were adults admitted to the ICU with a Glasgow Coma Score <8 due to hypoglycemia and persistent consciousness disorders after normalizing blood glucose levels. Patients with possible other causes of consciousness disorders, previous cognitive disorders, hypothermia <35 °C or circulatory arrest within 24 h after ICU admission, were excluded. Follow-up phone call was used to determine patients’ functional outcome using modified Rankin Scale (mRS) at a minimum of 1-year follow-up with mRS 0–3 defining good and mRS 4–6 poor outcomes.

**Results:**

Forty-nine patients were included. Causes of hypoglycemia were various, mainly including insulin or oral antidiabetic drugs abuse (65%) and neuroendocrine carcinoma (16%). Twenty (41%) patients died in the ICU, two (4%) patients further died and nine (18%) patients had a poor outcome at long-term follow-up. Five patients discharged from the ICU with mRS > 3 improved enough to be in the good outcome group 1 year later. Twenty-two (45%) patients underwent therapeutic limitation, mainly related to no expected hope for improvement. On multivariate analysis, only low mRS prior to ICU admission (OR 2.6; 95% CI 1.1–6.3; *P* = 0.03) and normal brain imaging (OR 7.1; 95% CI 1.1–44; *P* = 0.03) were significantly predictive of a good outcome. All patients (*n* = 15) who remained hypoglycemic >480 min had a poor outcome.

**Conclusion:**

Poor outcome was observed in about 60% of this population of hypoglycemic encephalopathy. However, some patients can recover satisfactorily over time.

## Background

Hypoglycemia is a common disorder, especially observed in diabetic patients. Neurological manifestations of hypoglycemia are wide and nonspecific including confusion, seizures, focal neurological deficits, stupor and sometimes coma [1–3]. Neurological deficits are usually fully reversible and non-life-threatening in case of short-duration hypoglycemia [4]. Although currently ill-defined, the hypoglycemic encephalopathy, which occurs during deep and/or prolonged hypoglycemia, is a sustained comatose state. Seizures and other neurological deficits may also be observed. The short- and long-term evolution of hypoglycemic encephalopathy, from full recovery to persistent vegetative state, remains yet poorly understood [1, 2, 5]. Several studies tried to identify prognosis factors but were limited by small sample size and/or short follow-up period [1, 2]. The hypoglycemic encephalopathy has been demonstrated to induce early lesions of the internal capsule that may secondarily reach the white matter [6, 7]. Their prognostic value is debated owing to the variability of the lesions [7] and the absence of correlation between severity of imaging lesions and patient prognosis [1, 6–8]. The objective of this multicenter study was to study causes, initial consequences and the vital and functional long-term prognosis of patients admitted to the ICU with prolonged neurological manifestations related to obvious hypoglycemia.

## Methods

### Study participants

We evaluated the data of patients admitted and treated in the ICU from July 1, 2004, to July 1, 2014, for prolonged hypoglycemic encephalopathy. Fifteen French adult ICUs were invited to participate in the study in September 2015. At each participating ICU, one investigator from the medical staff was responsible for identifying the patients using either administrative PMSI French database or local electronic medical records or both, collecting their data, and contacting them for follow-up news at least 1 year after the ICU admission. The diagnosis of prolonged hypoglycemic encephalopathy was considered in all patients >18 years old admitted in the ICU with (1) a Glasgow Coma Score lower than 8 on presentation, (2) hypoglycemic etiology of coma confirmed or highly likely with at least one measurement of blood glucose concentration <0.5 g/L on presentation, and (3) persistent consciousness disorders after normalizing blood glucose levels defined as persistent Glasgow Coma Score lower than 8 at least up to 24 h after ICU admission. Patients with possible other causes of consciousness disorders, previous cognitive disorders, hypothermia <35 °C or circulatory arrest within 24 h after ICU admission, or refusal to use their data were excluded.

### Data collection

General data included comorbidities, medical history, clinical findings, prehospital and admission blood glucose levels, Simplified Acute Physiological Score (SAPS II), need for and duration of mechanical ventilation, results of standard electroencephalogram (EEG), brain imaging, evoked potentials if any, main complications and length of ICU stay, decisions to withhold or withdraw care. Morbidity and mortality on ICU discharge were also recorded. The degree of disability was estimated with the modified Rankin Scale (mRS) before hospitalization (value on stable condition before the episode of hypoglycemia), at ICU discharge and, if available, at at least 1 year after ICU discharge [9]. The functional outcome was dichotomized into good (mRS 0–3) and poor (mRS 4–6). Scores range from 0 to 6: 0 indicating no symptoms at all; 1 indicating no significant disability despite symptoms, being able to carry out all usual duties and activities; 2 indicating slight disability, being unable to carry out all previous activities, but able to look after own affairs without assistance; 3 indicating moderate disability, requiring some help, but being able to walk without assistance; 4 indicating moderately severe disability, being unable to walk without assistance and unable to attend to own bodily needs without assistance; 5 indicating severe disability, being bedridden, incontinent and requiring constant nursing care and attention; and 6 indicating death. The mRS before hospitalization and on ICU discharge was evaluated using patients’ medical records including daily reports from doctors, nurses and physiotherapists and verified by the patients whenever possible at at least 1-year follow-up phone call. Finally, at least 1-year follow-up mRS was assessed by calling the patient, sometimes with the help of relatives. There was no structured interview, but all investigators were provided with the above-mentioned description of each mRS value; the reported mRS was the mRS of the patient at the time of phone call. Investigators were aware of the entire medical records of the patients they interviewed. To obtain these data, local investigators had to send previous information and consent form to the patient.

### Statistical methods

Descriptive results of continuous variables were expressed as mean and standard deviation or as median and interquartile range, depending on the normality of their distribution. Variables were tested for their association with prognosis by using Pearson’s Chi-squared test for categorical data and Mann–Whitney *U* test for numerical data. A multiple stepwise logistic regression model was established with any covariate with univariate significance of *P* value <0.10 eligible for inclusion in the model. The model was then further calibrated through Hosmer–Lemeshow testing.

### Ethical considerations

The study was approved by the Ethics Committee of the French Language Society of Critical Care Medicine. According to French law on noninterventional and retrospective studies, patients received written information about the study and non-opposition to their participation in the study was sought.

## Results

**Fig. 1:**
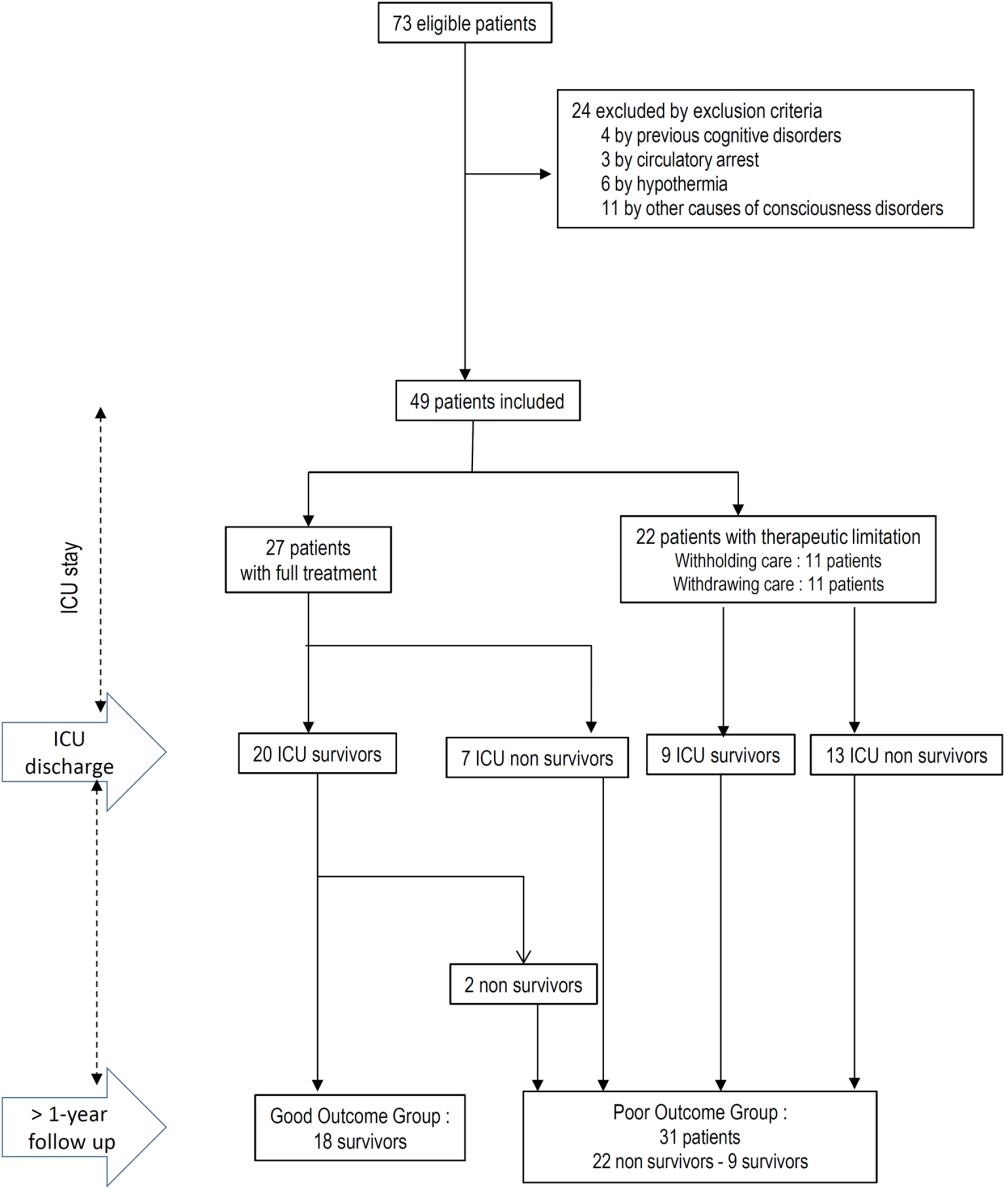
Flowchart

Among the 15 ICUs invited to participate, one reported that no patient fulfilled the inclusion criteria; nine admitted in the study period at least one patient that fulfilled the inclusion criteria and five provided no information. Seventy-three patients were eligible, and a total of 49 patients were finally included (Fig. 1). Their main characteristics are displayed in Table 1. Forty-one patients (84%) were hospitalized in the ICU within the first 24 h after the presumed onset of hypoglycemia and the eight remaining patients between 24 and 48 h. Insulin (*n* = 26), oral antidiabetic drugs (*n* = 5) or both (*n* = 1) were the cause of hypoglycemia in two-thirds of cases. Among the 46 patients who underwent mechanical ventilation, 10 were not sedated. The most frequent type of sedation was a combination of midazolam and sufentanil for 14 patients. Among other patients, 9 were sedated by propofol, 7 by the association of midazolam and fentanyl and 6 by midazolam only. Upon ICU admission, 20 patients (41%) were still hypoglycemic (glycemia <0.7 g/L).

**Table 1:** Baseline characteristics and outcome of patients with hypoglycemic encephalopathy

	All (*n* = 49)	Good outcome (mRS ≤ 3) (*n* = 18)	Poor outcome (mRS > 3) (*n* = 31)	*P* value
Male	27 (55)	9 (50)	18 (58)	0.28
Age (years)	55 [44–70]	52 [39–64]	56 [45–71]	0.11
Chronic alcohol abuse	17 (35)	5 (28)	12 (39)	0.23
Diabetes	29 (59)	8 (44)	21 (68)	0.01
SAPS II	55 [49–60]	54 [44–61]	55 [50–60]	0.75
mRS prior to ICU admission	1 [0–2]	0 [0–1]	1 [0–2]	0.03
Etiology				
Insulin and/or oral antidiabetics	32 (65)	12 (67)	20 (65)	0.89
Neuroendocrine carcinoma	8 (16)	2 (11)	6 (19)	
Alcohol abuse	2 (4)	1 (6)	1 (3)	
Adrenal insufficiency	1(2)	1 (6)	0 (0)	
Unknown	6 (12)	2 (11)	4 (13)	
Duration of hypoglycemia, min (*n* = 27)	480 [35–720]	45 [30–120]	660 [187–1283]	0.006
Initial glycemia (g/L)	0.21 ± 0.02	0.22 ± 0.03	0.20 ± 0.02	0.51
Glycemia on ICU admission (g/L)	1.01 ± 0.58	1.05 ± 0.16	1.50 ± 0.44	0.45
GCS				
Initial	4 ± 1	4 ± 1	4 ± 1	0.45
After glycemia normalization	5 ± 1	5 ± 2	5 ± 1	0.89
Seizures	8 (16)	6 (33)	2 (6)	<0.001
Temperature (°C)				
Initial	36.6 ± 0.2	36.7 ± 1.2	36.7 ± 0.5	0.91
Highest during first 24 h	37.9 ± 0.8	37.7 ± 0.8	38.0 ± 1.2	0.36
Initial arterial pH	7.38 ± 0.08	7.36 ± 0.12	7.40 ± 0.06	0.23
Initial lactate level (mmol/L)	2.54 ± 0.67	3.23 ± 1.36	2.10 ± 1.75	0.17
Time to final glycemia normalization (min)	180 [60–705]	480 [120–720]	180 [67–630]	0.78
Mechanical ventilation	46 (94)	16 (89)	30 (97)	0.55
Length of mechanical ventilation (days)	7 [3–12]	4.5 [2–8]	8 [6–13]	0.08
Normal EEG (*n* = 34)	1(3)	1 (10)	0 (0)	0.37
Normal brain imaging (*n* = 45)	25 (55)	11 (73)	14 (46)	0.009
Acquired ICU complications				
Pneumonia	25 (51)	8 (44)	17 (55)	0.28
Septic shock	8 (16)	3 (17)	5 (16)	0.78
ICU length of stay (days)	11 [5.5–17]	8 [4–14]	11 [7–17]	0.57
At least 1-year follow-up, days (*n* = 27)	840 [394–1768]	826 [407–1906]	1373 [388–1997]	0.93

Values are *n*(%), mean ± SD or median [IQR]

*GCS* Glasgow Coma Score, *SAPS II* Simplified Acute Physiological Score (version II)

Forty-five patients (92%) underwent brain imaging, 20 and 25 of them using MRI and CT scan, respectively. Most brain imaging examinations (*n* = 24) were performed on ICU admission with a median time of 0 day [0–5.5] (Table 2). Imaging was considered as normal in 25 patients. However, normal brain CT scans were significantly more frequent than normal MRIs (*P* < 0.001). In the 20 patients with abnormal imaging, lesions were bilateral in 16 of them. The most frequent abnormalities were located in the cortex (17%), the basal ganglia (15%) and the white matter (15%).

**Table 2:** Type and results of imaging

	All (*n* = 49)	Good outcome (mRS ≤ 3) (*n* = 18)	Poor outcome (mRS > 3) (*n* = 31)	*P* value
*CT scan*	25	10	15	0.15
Localization or type of lesions				
No lesion		10	13	0.5
Unilateral		0	1	1.0
Bilateral		0	1	1.0
Cortex		0	0	1.0
Basal ganglia		0	0	1.0
White matter		0	0	1.0
Ischemia		0	1	1.0
Edema		0	1	1.0
*MRI*	20	5	15	0.15
Localization or type of lesions				
No lesion		1	1	0.45
Unilateral		1	2	0.55
Bilateral		3	12	0.55
Cortex		2	7	1.0
Basal ganglia		2	6	1.0
White matter		2	6	1.0
Ischemia		1	4	1.0
Edema		0	2	1.0
*No imaging*	4	3	1	0.15

Data expressed in numbers

*CT* computed tomography, *MRI* magnetic resonance imaging

A standard EEG was performed in 34 patients (69%). Only one patient displayed a normal examination. The most frequently reported abnormality was a slowed brain electrical activity (22 out of 25 patients in the poor outcome group and 6 out of 9 in the poor outcome group) (*P* =0 .41).

Withholding life-sustaining care was decided for 11 patients (22%) after a median ICU stay of 7 [6–13] days, and withdrawing care was secondarily decided for two of them. Among the remaining patients, withdrawing care was decided as a first decision for 11 patients (22%) after a median ICU stay of 11 [6–15] days. No decision of therapeutic limitation was observed in the patients who reached a good outcome. Motivations included no hope for improvement (*n* = 19), expected limited quality of life (*n* = 8), expected limited autonomy (*n* = 6), heaviness of comorbidities and medical history (*n* = 5), severity of ICU complications (*n* = 3) and family request (*n* = 2). Nine patients who underwent care limitation, including three patients with withdrawal of care, survived to ICU discharge, all with a final poor outcome. The characteristics of patients with a decision of therapeutic limitation are compared to patients with no therapeutic restriction in Table 3. They displayed a significantly longer time under mechanical ventilation (*P* = 0.002) and ICU length of stay (*P* = 0.007) than patients with no therapeutic limitation.

**Table 3:** Characteristics of patients with or without a decision of care limitation

	No care limitation (*n* = 27)	Withholding/withdrawing care (*n* = 22)	*P* value
Male	13 (48)	14 (64)	0.38
Age (years)	53 [42–64]	58 [49–72]	0.15
Chronic alcohol abuse	20 (74)	9 (41)	0.04
Diabetes	14 (52)	15 (68)	0.38
SAPS II	53 [47–60]	57 [51–63]	0.17
mRS prior to ICU admission	1 [0–1]	1 [0–2]	0.40
Duration of hypoglycemia, min (*n* = 27)	90 [31–525]	720 [480–810]	0.07
Initial glycemia (g/L)	0.20 ± 0.13	0.22 ± 0.12	0.45
Glycemia on ICU admission (g/L)	1.42 ± 1.49	1.24 ± 1.18	0.86
GCS			
Initial	4 ± 1	4 ± 1	0.06
After glycemia normalization	5 ± 2	5 ± 1	0.55
Seizures	7 (26)	1 (4)	0.04
Temperature (°C)			
Initial	36.5 ± 1.2	36.9 ± 1.5	0.49
Highest during first 24 h	37.8 ± 1.2	38.1 ± 1.4	0.28
Initial arterial pH	7.36 ± 0.14	7.41 ± 0.09	0.39
Initial lactate level (mmol/L)	3.05 ± 3.04	1.81 ± 1.09	0.18
Time to final glycemia normalization (min)	390 [112–720]	180 [67–480]	0.42
Mechanical ventilation	24 (89)	22 (100)	0.24
Length of mechanical ventilation (days)	5.5 [2–8]	11 [7–16]	0.002
Normal EEG (*n* = 34)	1 (3.7)	0 (0)	0.41
Normal brain imaging (*n* = 45)	17 (63)	8 (36)	0.04
Acquired ICU complications			
Pneumonia	12 (44)	13 (59)	0.39
Septic shock	5 (19)	3 (14)	0.72
ICU length of stay (days)	7 [4–13]	14 [9–24]	0.007

Values are *n*(%), mean ± SD or median [IQR]

*GCS* Glasgow Coma Score, *SAPS II* Simplified Acute Physiological Score (version II)

Twenty patients (41%) died during ICU stay at a median time of 10 days [7–16] after admission, in relation to therapeutic limitation (*n* = 13), respiratory distress (*n* = 3), sudden cardiac arrest (*n* = 2) and brain death (*n* = 2).

**Fig. 2:**
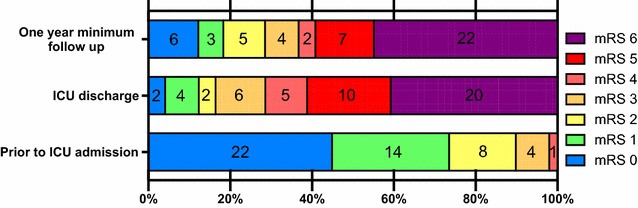
Distributions of patients modified Rankin Scale (mRS) prior to ICU admission, at ICU discharge and at a minimum of 1-year follow-up. mRS prior to admission was the mRS on stable condition before the episode of hypoglycemia. mRS 0 indicates no symptoms at all and mRS 6 indicates death. Good outcome is defined by mRS 0–3 and poor outcome by mRS 4–6

As shown in Fig. 2, there was a marked decline in functional status between admission and ICU discharge with a median increase in mRS of 4 [2–5]. Among survivors with poor outcome at ICU discharge (*n* = 15), one died 10 months later, six improved their outcome at at least 1-year follow-up with a median decrease in mRS of −2 [−2.5 to −1.75] including five patients who finally reached the good outcome group, and eight remained unchanged. Among the patients with good outcome at ICU discharge (*n* = 14), one died, seven further improved their outcome at 1 year with a median decrease in mRS of −2 [−2.0 to −1.0], whereas one patient deteriorated while remaining in the good outcome group (from mRS 1 to mRS2) and six remained unchanged. Finally, at 1-year follow-up, 18 patients (37%) displayed a good outcome with a median decrease in mRS of −1.5 [−2 to 0] as compared with ICU discharge.

Univariate analysis (Table 1) showed that diabetes, low mRS prior to ICU admission, short duration of hypoglycemia, onset of seizures and a normal brain imaging were associated with a better 1-year outcome. The topography of the lesions did not display any prognostic value.

**Fig. 3:**
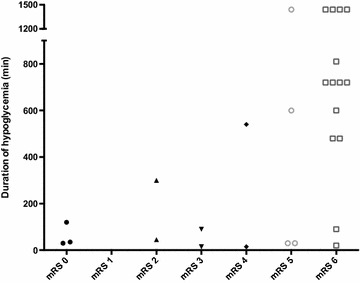
Modified Rankin Scale (mRS) at a minimum of 1-year follow-up according to the duration of hypoglycemia. Data were lacking for 22 patients

On multivariate analysis, only low mRS prior to ICU admission (OR 2.6; 95% CI 1.1–6.3; *P* = 0.03) and normal brain imaging (OR 7.1; 95% CI 1.1–44; *P* = 0.03) remained significantly predictive of outcome. The duration of hypoglycemia was not entered in the statistical analysis since values were unknown in 22 patients. However, as displayed in Fig. 3, all patients who remained hypoglycemic for 480 min or more evolved poorly while seven out of 12 patients with an exposition time to hypoglycemia below 300 min had a good outcome (*P* < 0.006).

## Discussion

In this multicenter study, we identified 49 patients hospitalized in several ICUs for a prolonged hypoglycemic encephalopathy over 10 years. A poor prognosis was observed in 31(63%) patients including 20 ICU non-survivors, two follow-up non-survivors and nine survivors (Fig. 1). Noteworthy, six patients who were discharged from the ICU with poor functional status improved their mRS at 1-year follow-up, including five who reached the good outcome group. Seeking to identify predictive variables of long-term outcome useful in daily practice, we found that low mRS prior to ICU admission and the absence of abnormalities on first brain imaging were significantly, although not strongly, predictive of outcome. Finally, the outcome of all patients with a long hypoglycemic duration was poor.

Available published data on prognosticators in prolonged hypoglycemic encephalopathy are based on cases series with a limited number of patients. To our knowledge, this study has the largest population of prolonged hypoglycemic encephalopathy requiring ICU hospitalization. Ikeda et al. [2] published a retrospective study of 165 patients to find predictors of 1-week good outcome in hypoglycemic encephalopathy. They included also patients with “hypoglycemic consciousness disturbance” which regained consciousness immediately after glucose administration, while our population was only constituted of severe patients requiring ICU because of a persistent coma over 24 h. Furthermore, 1-week follow-up was probably too short to fully evaluate this pathology. The retrospective study by Witsch et al. [1] included 15 patients from three centers with similar overall population’s characteristics regarding age, sex ratio, initial blood glucose levels, clinical severity and etiology than ours. Six of them died, two were lost to follow-up, five had a long-term good outcome and two had a poor outcome. These outcomes are consistent with the findings of the present study. However, the series was too small to identify outcome predictive factors.

As already known, hypoglycemic encephalopathy is more common in diabetic patients and diabetes was associated with poor outcome in univariate, but no longer in multivariate analysis. These data are consistent with findings of previous studies [1, 2, 7, 8]. They may be explained by a defective glucose counter-regulation in type-1 diabetic patients caused by frequent hypoglycemic episodes called “hypoglycemia-associated autonomic failure.” Symptoms due to the lack of glucose appear later and predispose to hypoglycemic encephalopathy [10].

Duration of hypoglycemia was a significant predictor of outcome in univariate analysis, but was not included in the multivariate analysis due to several missing data. For many patients, time between hypoglycemia onset and first glucose administration was impossible to precisely assess. This length was only available in 27 (55%) patients. Interestingly, a prolonged hypoglycemia was significantly associated with poor outcome, so that in our study, all patients who underwent hypoglycemia for at least 480 min had a poor outcome at 1-year follow-up (*n* = 15; see Fig. 3). In the paper by Ikeda et al. [2], a similar relationship was observed with a cutoff time for no good prognosis of about 48 h of hypoglycemia, which is longer than in the present study. These differences may be explained by both different criteria of good outcome and a very earlier outcome assessment than in the present study. Nevertheless, both studies strongly suggest that duration of hypoglycemia should be a major determinant of outcome.

Occurrence of seizures, most of them being observed early, was identified as a factor of good prognosis in univariate analysis. This issue has not been studied in the previous main hypoglycemic encephalopathy series [1, 2]. Our findings are not consistent with the results of a recent rodent hypoglycemic model study, showing that seizures and especially their frequency were associated with increased mortality [11]. Interestingly, a retrospective study about 425 patients with subarachnoid hemorrhage found that patients who presented early-onset seizures had a final good outcome, despite initial poor mRS [12]. Whatever the explanation of our findings, early-onset seizures should not be considered at least as adding more clinical severity in prolonged hypoglycemic encephalopathy.

Brain imaging has been evaluated as a prognosis tool [6–8, 13–16]. Yanagawa et al. [13] reported a case explaining how MRI had been useful to predict awakening and sequelae of their patient who underwent hypoglycemic encephalopathy. Johkura et al. [6] prospectively studied early diffusion MRI as a predictor of short-term outcome in 36 patients with hypoglycemic encephalopathy. The absence of lesions on the first early diffusion MRI was associated with good outcome. Conversely, Witsch et al. [1] did not find any prognostic value in imaging, but this retrospective study recruited 15 patients, of them only three had MRI. In the present study, lesion localization at first imaging (including MRI and CT scan) was not predictive of outcome but normal early imaging that was mostly observed in brain CT scans was considered as a factor of good outcome.

In contrast to Ikeda et al. [2], the initial serum glucose concentration, the body temperature during hypoglycemia and the blood lactate concentration were not related to the final patient outcome in our study. This discrepancy may be explained by several reasons. Ikeda et al. [2] defined good and poor outcomes groups using Glasgow Outcome Scale (GOS) and poor outcome included patients with GOS from 1 to 4. Thereby, patients with moderate disability but independent in daily living were considered as poor outcome patients, while they would have been included in our good outcome group. Furthermore, even if Ikeda et al. [2] included 165 patients, lactate measurement was available in only 19 of them.

Therapeutic limitation was decided in 22 patients. All of them further displayed a poor outcome. Decisions were made in seven patients within 1 week after ICU admission, with a minimal time of 2 days (*n* = 1). Decisions of therapeutic limitation were mostly based on the clinical severity of neurological insult and the expected absence of significant improvement with time. A similar reasoning is usual in anoxic–hypoxic encephalopathy. However, mechanisms of brain damage are different [17, 18], so that clinical prognostic tools used for decades in hypoxic encephalopathy have yet to be validated in hypoglycemic encephalopathy. Keeping in mind that available evidence about prognosis of severe hypoglycemic encephalopathy is scarce, therapeutic limitation could constitute an important bias, leading to “self-fulfilling prophecies” [19]. On the one hand, patients with therapeutic limitations had a longer time under mechanical ventilation and stay in the ICU, a longer duration of hypoglycemia, and survivors did not improve their outcome over time as compared with not limited patients. On the other hand, the expected time for clinical improvement appears to be long as we observed that five patients with no therapeutic limitation discharged from the ICU with mRS > 3 improved enough to be later in the good outcome group. Taken together, these findings suggest that decisions to withhold or withdraw care should be taken very cautiously, obviously not prematurely after ICU admission. Ten to fifteen days with no clinical improvement could be a minimal threshold, based on the minimal time from admission to decision of therapeutic limitation among the patients who survived their ICU stay. In addition, normal brain imaging, especially using RMI, could help to further postpone a decision to limit care.

Our study has several limitations. Several data such as duration of hypoglycemia were missing. Selection biases cannot be excluded regarding the non-responding invited ICU centers. Furthermore, initial glycemia was mostly assessed (94%) using capillary glucometers that are less reliable than laboratory measurements using blood venous samples. Our population was small due to the scarcity of this disease, and a larger population would have allowed a more robust multivariate analysis. In addition, several patients did not undergo brain MRI, a more sensitive tool than CT scan, and four of them had no imaging at all. Continuous EEG monitoring, never performed here could have been more relevant to rule out subclinical status epilepticus or better delineate patterns with prognosis value. Finally, mRS assessment was not based on a standardized questionnaire, which could have led to possible mistakes.

## Conclusion

This multicenter study confirms that prolonged hypoglycemic encephalopathy is a severe condition leading to a poor long-term outcome. Factors of good outcome were a low mRS prior to admission and normal brain imaging, while the hypothesis of a long duration of hypoglycemia as a predictor of poor outcome is suggested. However, some patients with severe impairment at ICU discharge may improve later, suggesting that therapeutic limitation should not be decided early. Finally, the present findings offer prognostic tools, which need to be further delineated in larger, prospective investigations.

## Abbreviations

CT: computed tomography
EEG: electroencephalogram
GOS: Glasgow Outcome Scale
ICU: intensive care unit
MRI: magnetic resonance imaging
mRS: modified Rankin Scale
SAPSII: Simplified Acute Physiological Score (version II)
